# Refugee and Asylum Seeker Trauma Informed Care Training for Oral Healthcare Professionals in NSW, Australia

**DOI:** 10.3389/froh.2022.907758

**Published:** 2022-05-31

**Authors:** Siobhan Kelton, Kanchan Marcus, Graeme Liston, Angela Masoe, Woosung Sohn

**Affiliations:** ^1^Centre for Oral Health Strategy, NSW Ministry of Health, North Sydney, NSW, Australia; ^2^Population Oral Health, Faculty of Medicine and Health, The University of Sydney School of Dentistry, Sydney, NSW, Australia

**Keywords:** refugee, asylum, oral health, trauma informed care (TIC), healthcare professionals

## Abstract

People from refugee and asylum seeker (RAS) backgrounds who have re-settled in Australia experience inequitable health outcomes. As a result, people from RAS backgrounds need access to culturally safe and responsive care. To provide this care, oral health professionals must understand how experiences of trauma influence a patient's oral health. The aim of this study was to highlight the lessons learnt from providing trauma informed care (TIC) to oral health professionals in New South Wales (NSW). TIC is a model that emphasises trust, patient safety, choice and empowerment to foster healthcare equity. This study was designed and piloted by the Centre for Oral Health Strategy (COHS), NSW Ministry of Health in partnership with NSW Refugee Health Service, local Multicultural Health Services, and four Local Health Districts (LHDs): Hunter New England, Mid-North Coast, Murrumbidgee and Illawarra Shoalhaven. Pre and post TIC training surveys were distributed to oral health professionals. This captured baseline versus intervention data to understand their knowledge of TIC. Seven training sessions were provided by NSW Refugee Health Service in four LHDs. A total of 152 participants attended a TIC training session, 106 participants completed the pre-survey, and 67 participants completed the post-survey. At baseline, only 50% of staff reported confidence in delivering TIC care to RAS populations. After the intervention, 97% of staff reported feeling extremely, very, or somewhat confident in understanding and delivering TIC. Findings demonstrate that TIC training can support oral health professionals to provide culturally safe and responsive care to people from RAS backgrounds.

## Introduction

Global disparities in the access and utilisation of oral healthcare services are prevalent and widely cited [1–3]. In particular, people from refugee and asylum seeker (RAS) backgrounds experience difficulties with accessing both the general healthcare and oral healthcare in re-settlement countries [4]. Challenges and barriers experienced in accessing oral healthcare include affordability, communication, accessibility issues within the healthcare system and negative provider interactions, among others [4]. The consequence of difficulties with access to oral healthcare is poorer oral health status of these RAS populations compared to that of general non-RAS populations [4].

In Australia, a growing number of RAS people are re-settled in rural and regional areas of New South Wales (NSW) [5]. Over a period of 5 years, 2015–2020, there were 30,531 humanitarian arrivals in NSW, with the majority residing in South Western Sydney (19,463) and Western Sydney (4,179) [5]. In 2011, the first state-wide plan (NSW Refugee Health Plan 2011–2016) dedicated to improving the health and wellbeing of refugees and people with refugee-like experiences who have settled in NSW was published by the NSW Ministry of Health [6]. The plan identifies oral health as a priority health issue for RAS populations. Furthermore, the plan recognises that a model of care needs to be developed to improve access to oral health services.

To reduce the oral health inequities experienced by RAS populations, targeted interventions and programs are required. For these approaches to be effective, culturally safe and responsive, care must be provided to people from RAS backgrounds. Health professionals must be supported if they are to provide this care. For instance, a meta-synthesis study of healthcare services for refugees demonstrated that the attitudes of healthcare professionals influence how care is received by people from RAS backgrounds [4]. This review highlighted several barriers to the provision of healthcare, including cultural misunderstandings, miscommunication, limited cultural competency and trust between healthcare providers and patients from RAS backgrounds [4]. Furthermore, a growing body of evidence on cultural competency substantiates the need for an organisational commitment to cultural competence, strong partnerships with refugee communities and the integration of language and culture in service delivery [7]. A scoping review that analysed 26 articles on cultural competence in refugee populations identified the need for context-specific solutions given the heterogeneity in countries, diversity in populations and types of primary service settings [7].

To improve oral healthcare for RAS populations, the Centre for Oral Health Strategy (COHS) NSW designed and implemented a trauma informed care (TIC) based training program. Traumatic events, including war, torture, imprisonment, physical and/or sexual assault, can affect an individual's physical and emotional health and wellbeing. Consequently, a trauma informed approach to care acknowledges how trauma can affect a person's health and wellbeing, patterns of accessing care, trust, communication and behaviour in clinical settings [8]. With this understanding in mind, service providers can enhance models of care to better meet the needs of the patient. Thus, the TIC model is person oriented, collaborative and considerate of individual's cultural, historical, emotional and psychosocial factors, which guide provider interactions [9].

A previous study reported the significant need for health professional training to enhance awareness of refugee-specific needs, including the need for collaboration and the establishment of mutual expectations between healthcare providers and, more broadly, the re-settlement community [10]. While this study highlighted the beneficial impact of TIC in supporting RAS populations, this study did not specifically focus on oral healthcare professionals. Notably, the incorporation of trauma informed care (TIC) into oral health service delivery for RAS populations is largely absent in the literature. Thus, a pilot project, which included the provision of TIC training for oral health professionals, was an opportunity to better understand the oral healthcare provider experiences of TIC with RAS populations in Australia. The purpose of this article is to contribute to the evidence base for TIC by examining the key lessons learned from providing trauma informed care training to oral health professionals in NSW.

## Methods

In 2021, the COHS received funding from the Community Care and Priority Populations Unit, Health and Social Policy Branch to improve access to oral healthcare and preventive information for people from RAS backgrounds. This pilot project was implemented between March and June 2021 and was delivered in partnership with NSW Refugee Health Service and four rural NSW Local Health Districts (LHDs): Hunter New England LHD (HNELHD), Mid-North Coast LHD (MNCLHD), Murrumbidgee LHD (MLHD) and Illawarra Shoalhaven LHD (ISLHD). A project management team with representatives from each LHD was established to support the implementation of this project.

As part of this project, trauma informed care (TIC) in-service training was delivered to oral health teams in four LHDs in rural NSW. The training was delivered by the Director of the NSW Refugee Health Service, a public health physician. In total, seven 1 hour training sessions were delivered to 152 oral health professionals in four LHDs. The sample size was not statistically calculated, as descriptive analysis is undertaken to gauge an understanding of the pilot programme. In this study, “oral health professionals” include dental practitioners, such as dentists, oral health therapists, dental therapists and non-clinical staff, including dental assistants and administrators. Due to coronavirus disease 2019 (COVID-19) restrictions, oral health staff who were available to support the RAS program participated in the virtual training rather than in-person.

Trauma informed care is a model that emphasises trust, patient safety, choice and empowerment to help foster healthcare equity [9]. TIC was developed by Harries and Fallot [11] using trauma theory to design service systems: new directions for mental health services in 2001. For this NSW pilot study, TIC training identified the types of trauma that refugees might have experienced, including persecution, war, kidnapping, imprisonment, torture, loss of family and friends, witnessing atrocities and sexual violence. Further training discussed the potential effects of this trauma in a clinical setting. For example, intrusive memories may be triggered when the patient is receiving dental care. Patient fear and hostility can, therefore, be understood as a potential response to trauma. As a result, trauma informed practitioners focus on providing a physical and emotionally safe environment for patients. These practitioners will ensure that communication is open and respectful and, where possible, gives choice and control to patients through a collaborative approach to healthcare that supports the patient's goals.

To evaluate the effectiveness of the training, pre and post TIC training surveys were distributed to participants. The surveys were developed by the COHS in consultation with Refugee Health Services. Consultations with the project management team led to survey refinements. TIC training surveys were distributed to oral health professionals to obtain baseline vs. intervention data to understand their knowledge of trauma informed care. The surveys utilised a Likert scale measure to record participant responses. The survey asked respondents to rank their knowledge of the health issues affecting refugees on a scale of “poor” (1) to “excellent” (5). To ascertain the confidence of oral health staff in providing trauma informed care, the survey asked: “How would you rate your confidence in providing TIC to people who are RAS?” with answers rated between “extremely confident” to “not at all confident.” The survey question: “How would you rate your knowledge of each of the following areas” also probed “definition of the terms refugee and asylum seeker;” “refugee and asylum seeker eligibility for oral health services;” “why oral health status is poor in the refugee and asylum seeker community” and “techniques for providing TIC.” These were ranked from “poor” (1) to “excellent” (5). An optional, open-ended question was included at the conclusion of the post-survey to capture free responses about the TIC from participants. The survey question: “Please tell us if you have any other comments about this training?.” The question allowed participants to reflect and elaborate on the responses provided to the close-ended questions. In total, 16 participants responded to this question. Fifty three respondents skipped the question.

Descriptive analysis for pre and post survey data was undertaken for this project. This methodology is commonly utilised within the healthcare field to summarise findings about a specific program or population [12]. This is relevant to this project because the training was provided to oral health professionals that work within the NSW Health System. The frequency and distribution of responses for pre and post survey ordinal variables were conducted. The average mean was calculated for each survey response. Open-ended responses of the survey were analysed using content analysis, which incorporated an iterative approach [13]. In this process, verbatim responses were coded into meaningful categories to gauge feedback about the TIC pilot program [13]. Codes were sorted into “beneficial impacts” and “suggestions/recommendations,” which supported the quantitative analysis of the pre/post survey data. Conceptually, this study adopts a TIC lens; however, a theoretical framework was not utilised, considering that this pilot project was exploratory in nature. Future evaluations of this project will incorporate qualitative and theoretical components, which will further enable thematic analysis of this program. Excel was utilised for all the data coding and analysis.

Oral health professionals consented to participate in the pilot program activities as part of their employment requirements. However, participation in the pre and post training survey was voluntary. This is a health professional development and quality improvement report; therefore, ethics approval is waivered.

## Results

Out of 152 people who attended the training, 106 participants completed the pre-training survey. However, only 69 participants completed the post-training survey. This equals a response rate of approximately 70% for pre and 45% for post training survey, respectively.

There was a significant improvement in respondents' knowledge regarding health issues affecting RAS populations. The mean rank for each question in the pre-survey was between 2.31 and 3.16. This indicates that most respondents had “fair” to “good” understanding of the health issues that affect people from RAS backgrounds (Table 1). After attending the TIC training, 69 respondents had an increased mean rank for each question (4.21–4.26). That is, knowledge about RAS terminology improved from 10.6% at pre survey to 42.7% post survey. Understanding why oral health status was poor in RAS populations was only 30.8% “good” to “excellent” at pre-survey and 83.9% post-survey for the same. Only 2.9% reported “excellent” at pre-survey for techniques for providing TIC, but this jumped to 41.2% at post-survey. In total, 75% of respondents felt that their understanding of trauma informed techniques was good to excellent after training.

**Table 1:** Demographic characteristics of pilot trauma informed care (TIC) intervention.

		**Pre-survey**	**Post-survey**
		***N*** **= 104**	***N*** **= 67**
Specialty	Administrator	10	6
	Dental Assistant	52	30
	Dental Officer	6	3
	Oral Health Therapist	13	12
	Dentist	6	8
	Healthcare Worker	4	4
	Coordinator	5	2
	Manager	7	2
	Not identified	1	0
Local Health District	Hunter New England	10%	10%
	Mid North Coast	48%	58%
	Illawarra Shoalhaven	41%	32%
	Murrumbidgee	1%	0
**How would you rate your knowledge of each of the following areas?**			
The definition of the terms “refugee” and “asylum seeker”	1- Poor	4.8%	0
	2	22.1%	0
	3 - Fair	35.6%	17.7%
	4	26.9%	39.7%
	5 -Excellent	10.6%	42.7%
	Mean score	3.16	4.25
Refugee and asylum seeker eligibility for oral health services	1- Poor	12.5%	0
	2	22.1%	0
	3 - Fair	34.6%	17.7%
	4	21.2%	44.1%
	5 -Excellent	9.6%	38.2%
	Mean score	2.93	4.21
Why oral health status is poor in the refugee and asylum seeker community	1- Poor	4.8%	0
	2	23.1%	0
	3 - Fair	34.6%	16.2%
	4	29.8%	41.2%
	5 -Excellent	7.7%	42.7%
	Mean score	3.13	4.26
Techniques for providing trauma informed care	1- Poor	22.1%	0
	2	39.4%	1.5%
	3 - Fair	26.9%	23.5%
	4	8.7%	33.8%
	5 -Excellent	2.9%	41.2%
	Mean score	2.31	4.15

**Figure 1:**
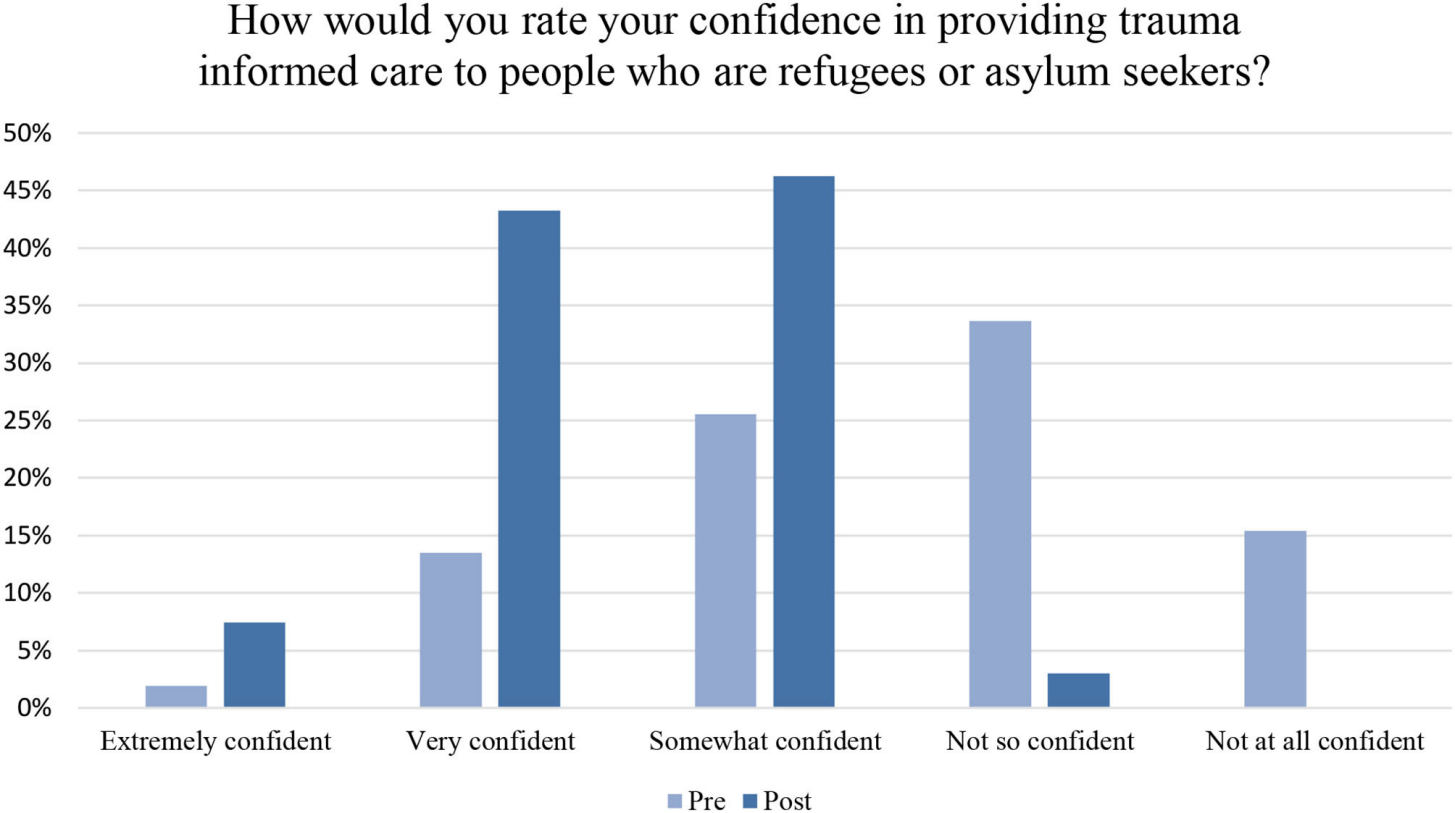
Pre/post training survey.

The second part of the survey asked respondents to rate their confidence in providing trauma informed care to people from RAS backgrounds. A total of 97% respondents indicated that they felt “extremely,” “very,” or “somewhat” confident in delivering TIC. Only 2 respondents (3%) said that they felt “not so” confident and no respondents indicated that they felt “not at all” confident (as opposed to 15.4% in the pre-survey) (Figure 1). At the point of completion, the majority (88.5%) indicated that their knowledge of trauma informed techniques was poor to average. Further, 49% of respondents also said they were “not so” or “not at all” confident in providing TIC to people from RAS background (Figure 1).

**Figure 2:**
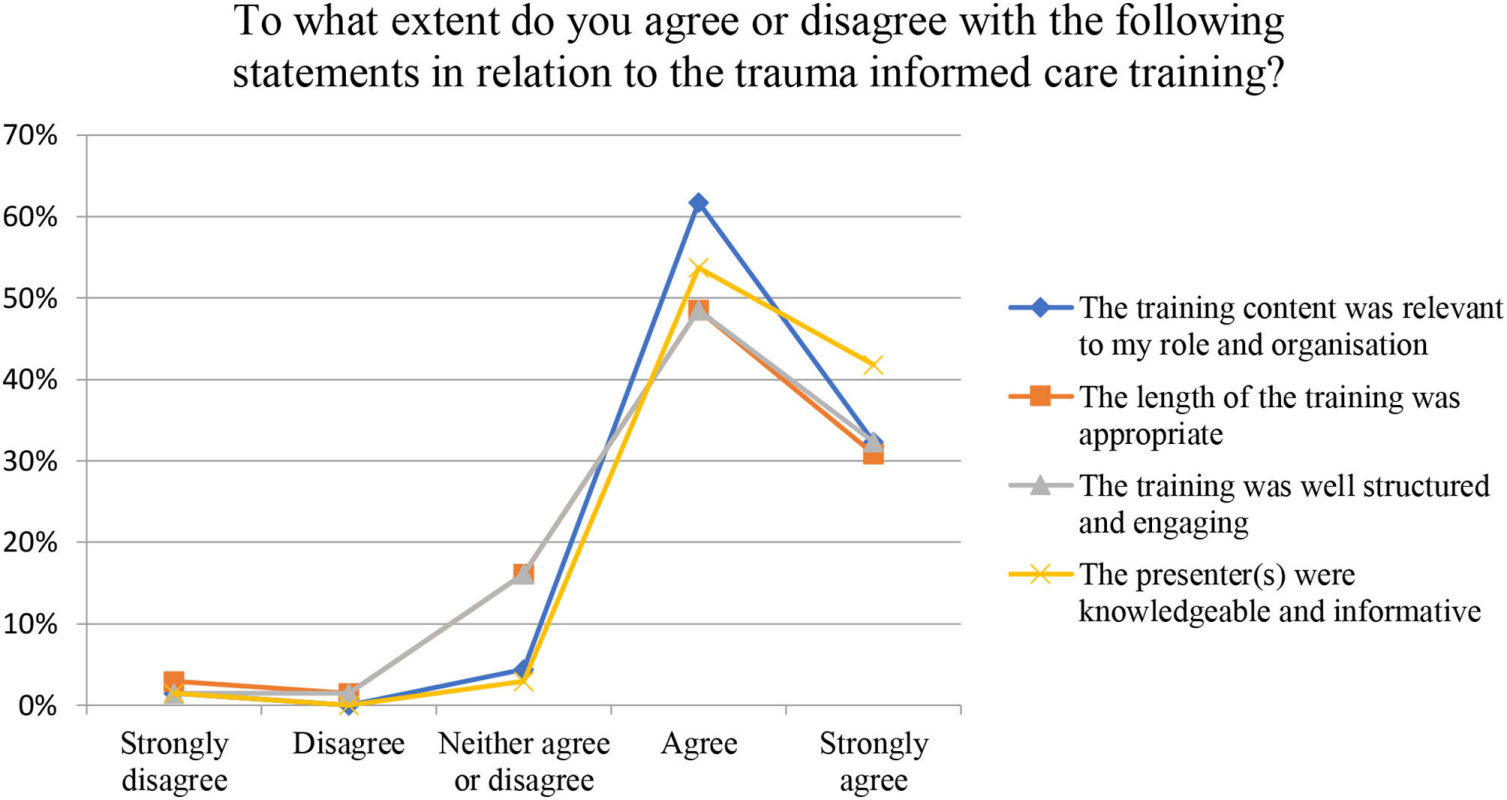
Post training trauma informed care (TIC) survey.

Sixteen participants completed qualitative descriptions to the TIC post-training survey. Content analysis revealed two key concepts: first, the beneficial impact of TIC and second, areas for improvements and suggested recommendations to enhance the delivery of the training. One participant stated that “… it is important for us [oral health professionals] to understand the trauma that patients may have suffered to allow us to be more compassionate and understanding of their needs.” Another participant stated that the training helped them feel “empathy and compassion” for patients that have a refugee or asylum seeker background. For one dental practitioner, the training helped them to understand why a history of trauma may mean that patients feel uncomfortable for attending a dental clinic. However, another dental practitioner said that the training required more practical “tools or tips” that could be implemented in a clinical setting. Three participants stated that the training was not well structured or engaging, but thirteen participants agreed that the training was relevant to their role and organisation, that the length of training was appropriate, that the training was structured and engaging and that the presenter was knowledgeable and informative (Figure 2).

## Discussion

This study reports lessons learned from the delivery of TIC training to oral health professionals employed in NSW public dental services. Results from this study demonstrated that TIC training enhances oral health professional's capacity to provide culturally safe and responsive care to people from RAS backgrounds. There was a notable increase in the proportion of the participants who reported “extremely,” “very,” and “somewhat” confident in providing trauma informed care to patients. Participants reported feeling more “compassionate” and “empathetic” toward people from RAS backgrounds. This is significant because in a recent review on the concept and meaning of empathy in healthcare settings, it was found that empathy improves the therapeutic relationship between providers of healthcare and their patients and leads to better health outcomes [14]. Despite the importance of empathy in the clinical setting, this review also reported that a high number of health professionals find it difficult to develop empathy in relation to the patient [14].

This study aligns with current evidence and supports the need for TIC training to be scaled statewide [10]. In a study by Raja and colleagues, core TIC principles include patient-centered care, understanding trauma and biases, inter-professional collaboration and screening [8]. This study further proposed a framework for improving patient-provider interactions when providing medical care [8]. Importantly, TIC requires cultural competency skills for staff, in order to provide adequate healthcare to the population [8]. However, few studies have identified the importance of TIC with oral healthcare professionals, particularly in the Australian context. For example, one literature review explored TIC with mental health nurses in Australia. This study highlighted the benefits of TIC, which incorporated person-centered therapeutic relationships, choice and autonomy that improved consumer care experiences [15]. Further research and evaluation is required for TIC with oral healthcare professionals in providing care to RAS populations.

This study has a few limitations. First, the pre/post training surveys were self-reported and thus, clinical outcomes are not tested and unknown. That is, the pilot program did not conduct practical examinations to determine the extent to which health professional application of TIC was successful. TIC training may slightly differ between LHDs in the delivery and contents of training and if delivered by various people. Methods to minimise this variance would be required, such as workshops for teams delivering TIC. Study of TIC in the Australian primary healthcare setting is largely missing in the literature and, thus, effectiveness of TIC needs to be further evaluated. While this study provides evidence of oral health professional improvements in knowledge and confidence, further translational study is required to establish culturally safe and patient-centered oral healthcare for RAS populations. If this evaluation was to be repeated, TIC training would need to more rigorously evaluated. This may involve undertaking detailed interviews/focus groups with participants at pre-determined intervals (for example, 3, 6, and 12 months). Despite these limitations, TIC has the potential to improve both the provision and receipt of care.

As a result of this pilot study, three key learnings are evident. First, a more rigorous evaluation of the TIC training must be conducted. Second, cultural competence validated tools can be incorporated to the pre/post survey designs to add rigor to data collected. Third, format of the training should also be re-examined. Due to COVID-19 restrictions, the training was delivered online. There were some advantages to delivering the training online: the training could be recorded and there were no limits on the number of participants who could attend the training. However, whether in-person training could be more engaging needs to be explored. An in-person format could allow for more interactive content, including role-play, which could further improve the confidence of oral health professionals in providing TIC. This format may be disadvantageous because it is more resource/time intensive, particularly if travel to regional and remote areas is required. Alternatively, an online training module hosted on an electronic learning (eLearning) system could be feasible. My Health Learning, NSW Health's eLearning system [16], provides high-quality, self-managed online learning for NSW Health staff and was introduced to standardise the delivery of education and the assessment of staff knowledge and skills across NSW Health. It is important that these options are explored as part of the next phase of this project.

## Conclusion

The findings from this study demonstrate that trauma informed care training can support oral health professionals to provide culturally safe and responsive care to people from RAS backgrounds. As the training can improve both the provision and acceptance of care, a state-wide roll out of the training should be considered. However, the format of the training should be re-examined and a more rigorous evaluation plan of the training must be in place.

## Data Availability Statement

The original contributions presented in the study are included in the article/supplementary material and further inquiries can be directed to the corresponding author.

## Ethics Statement

Ethical review and approval was not required for the study on human participants in accordance with the local legislation and institutional requirements. The Ethics Committee waived the requirement of written informed consent for participation.

## Author Contributions

This study was conceptualised by the Centre for Oral Health Strategy, NSW Ministry of Health, GL and Local Health Districts. SK and KM drafted the initial manuscript, which was constructively edited by AM and WS. All the authors edited the article and agreed to this final version of the manuscript.

## Conflict of Interest

The authors declare that the research was conducted in the absence of any commercial or financial relationships that could be construed as a potential conflict of interest.

## Publisher's Note

All claims expressed in this article are solely those of the authors and do not necessarily represent those of their affiliated organizations, or those of the publisher, the editors and the reviewers. Any product that may be evaluated in this article, or claim that may be made by its manufacturer, is not guaranteed or endorsed by the publisher.
